# Boosting Student’s Motivation through Gamification in Physical Education

**DOI:** 10.3390/bs13020165

**Published:** 2023-02-14

**Authors:** Víctor Javier Sotos-Martínez, Juan Tortosa-Martínez, Salvador Baena-Morales, Alberto Ferriz-Valero

**Affiliations:** 1 Department of General and Specific Didactics, University of Alicante, 03690 Alicante, Spain; 2 Faculty of Education, Valencian International University (VIU), 46002 Valencia, Spain; 3 HEALTH-TECH Research Group, Department of General and Specific Didactics, University of Alicante, 03690 Alicante, Spain; 4 EDUCAPHYS Research Group, Department of General and Specific Didactics, University of Alicante, 03690 Alicante, Spain

**Keywords:** methodology innovation, elementary education, primary school, gamified learning, motivational regulation, quality education, SDG 4

## Abstract

Students are becoming less motivated towards current education. For this reason, teachers are investigating several innovative methodologies to learn how they affect student motivation, such as gamification. The purpose of this study was to analyze the effects of gamification on the motivation of elementary physical education students. A total of 72 elementary school students from two different Spanish elementary schools participated (38 boys and 34 girls), separated into a gamified group (*n* = 35) and a control group (*n* = 37). Ten gamification sessions were performed using a technological app called ClassDojo. The gamified proposal was based on both a PBL model and an MDA model. A questionnaire, “Motivation Questionnaire in Physical Education” (CMEF-EP) was used to measure the motivation of the students before and after the intervention. An increase was observed in all the variables for the gamified group: intrinsic motivation (*p* < 0.001), identified regulation (*p* < 0.001), introjected regulation (*p* = 0.001), and external regulation (*p* = 0.002), except for the amotivation (*p* = 0.120). No changes were observed in the control group. A significant interaction effect over time was seen only for intrinsic motivation for the gamified group versus the control group (F(1) = 5.263; *p* = 0.025; η^2^ = 0.070). The results show the efficacy of gamification to increase the motivation of elementary physical education students. However, it does not decrease amotivation. This will enable the subject to contribute to achieving the United Nations’ proposed Sustainable Development Goal 4, which is to ‘Improve Quality Education’.

## 1. Introduction

Motivation is a key point to achieve successful learning [1], but a great challenge in the educational context as many children that present high amotivation levels [2], make the teaching-learning process difficult [1]. Thus, designing appropriate strategies to increase student’s motivation is a priority for education systems. In order to understand motivation, self-determination theory (SDT) [3] is a useful macro theory which divides motivation into different states within an autonomous or controlled motivation continuum: intrinsic motivation, when the activity makes students feel comfortable and satisfied; extrinsic motivation, when the activity is useful to achieve external recognition; and amotivation or lack of motivation [1]. In addition, extrinsic regulation is divided into four categories according to the nature of the motivation reasons: integrated regulation, day-to-day activity or daily task; identified regulation, activity carried out for the benefits it presents; introjected regulation, feeling guilty for not taking part in the activity; and external regulation, participating in an activity for achievement of rewards [4].

In order to improve the teaching-learning process and increase motivation, researchers are searching for innovative methods of learning, such as gamification [5,6]. This term has been defined based on several different perspectives. One of the most widely used definitions describes it as items from games used in other environments (e.g., education) [7]. Gamification was later defined as attempting to resemble an activity to a game [8] or as a process to improve the experience of an activity through play to increase the value perceived by users [9]. Gamification has already been widely used in other disciplines such as marketing, healthcare, human resources, training, environmental protection and well-being [10], or other curricular and academic fields such as higher education [11]. When implementing a gamified strategy, the choice and proper use of gamification models must be considered. There are two general models of implementation for gamification. The PBL model (Points-Badges-Leaderboards) is used when providing rewards and punishments to participants according to their behavior. These rewards or punishments can be presented in many ways such as increases and reductions in points or actions, which can serve as an advantage. The MDA model is based on mechanics, a rule description, logic and events in the history tab of the class; dynamics, mechanical and dynamic results description of actions; and aesthetics, which describe students’ emotional reactions [12,13]. These two models can be combined in a single intervention.

Physical education (PE) is one of the subjects that allows the greatest use of games due to the playful nature of the activities [14], yet there is little scientific evidence about the benefits of gamification in PE, and most studies present methodological weaknesses. In secondary education, Monguillot-Hernando et al. [15] found increments in motivation after a gamification intervention. Similarly, Martín-Moya et al. [5] found increases in motivation and commitment levels of the students. However, these studies did not differentiate the motivation type. Sotos-Martínez et al. [6] applied a gamified intervention through information and communication technologies using Classcraft^®^, and observed an improvement in intrinsic motivation as well as a decrease in amotivation. In the same direction, Segura-Robles et al. [16] prepared a gamification combined with a flipped classroom, finding higher intrinsic motivation values, although these results could be influenced by the flipped-classroom method implanted. Nevertheless, other authors have pointed out that increases in extrinsic motivation could also appear when gamification is used [17,18]. In elementary education, research is even more scarce. Some authors have observed that, through a gamified proposal, students improved their intrinsic motivation by making use of various badges, points, rewards and narrative [19]. Similarly, other authors have used a gamification based on Just Dance with PBL and MDA models [20], showing increments in the students’ basic psychological needs (BPNs), which were correlated positively with intrinsic motivation [1]. Another research found that amotivation was reduced after applying a gamified proposal in elementary school students [21].

Considering the limited number of studies, more research is needed to establish how gamification in elementary education affects motivation of PE students. For this reason, the objective of the present work was to analyze the impact of a gamified intervention in elementary school PE classes compared to a non-gamified intervention on students’ motivation variables.

Based on the preceding literature, the hypotheses for this research are as follows:

- H1. A gamified intervention in elementary PE will produce higher intrinsic motivation improvements in the gamified group compared to the control group.
- H2. A gamified intervention in elementary PE will not produce changes in extrinsic motivation values in the gamified group.
- H3. A gamified intervention in elementary PE will produce lower amotivation levels in the gamified group compared to the control group.

## 2. Materials and Methods

### 2.1. Study Design

For this study, a natural experimental design with a cluster randomized sampling was chosen. The participants were divided into two large groups, the control group (CG) and the gamification group (EG). In order to do this, two classes from the same education center for each different level were chosen, placing one class in the EG and the other in the CG, after random sampling in clusters, maintaining one each of EG and CG per level of education. The intervention of the EG followed a teaching method based on a gamified proposal, while in the CG a traditional methodology was applied [22].

### 2.2. Participants

The sample of students who participated in the research consisted of 72 students (9–11 years old, 52.8% boys and 47.2% girls). Two Spanish elementary schools agreed to participate in the present study. A total of 11 students were excluded from the sample as they did not meet any of the following requirements: participation in at least 80% of the sessions, appropriate completion of the questionnaire, good physical condition to enable participation in the sessions and properly completed informed consent to participate in the study. Prior to the beginning of the intervention, an informed consent was provided to be returned correctly completed by the student’s tutor, according to principles of the Helsinki Declaration (1975). The validation of this research was carried out by the ethics committee of the University of Alicante (UA-2022-05-24).

### 2.3. Instruments

The questionnaire used was the motivation questionnaire in physical education (CMEF-EP) [23], which is composed of 18 items that measure intrinsic motivation, identified regulation, introjected regulation, external regulation and amotivation (Table 1). However, integrated regulation is not measured in this questionnaire. The validation of the questionnaire was carried out with 333 Spanish elementary school students (10–12 years). The results of the adjustment index values in all cases were acceptable (χ^2^/df = 1.80; RMSEA = 0.04; CFI = 0.92; GFI = 0.93; TLI = 0.91; SRMR = 0.05) (Leo et al., 2016). In the current research, Cronbach’s alpha analysis showed 0.826 for intrinsic motivation, 0.848 for identified regulation, 0.814 for introjected regulation, 0.885 for external regulation and 0.787 for amotivation.

### 2.4. Process

The current research was conducted during the 2021/22 academic year in the subject of PE. The intervention consisted of the implementation of a 10-lesson proposal, spread over six weeks. The implementation within the EG was based on a gamified proposal, where the teacher controlled the sessions through gamification so as to decrease the uncontrolled variables that could influence the measured results. The CG sessions were also conducted by official teachers for the same reason. These teachers were taught how to implement a gamified intervention in their sessions correctly. In order to identify the change produced by the intervention, a questionnaire based on motivational regulations had to be completed by the participants before the first session and after the last. The gamified proposal included the use of information and communication technologies (ICT) based on the gamified pedagogical tool ClassDojo^®^ (https://www.classdojo.com/es-es/). Depending on the educational level and the school program, even by using this tool, students developed specific contents, and there was no difference when comparing classes at the same level regardless of the group (EG or CG). The methodology used was identical for both groups, with an additional one initial and final minute to provide the gamification narrative. ClassDojo is a tool which uses positive behavior points or negative behavior as the main concept; in this way it is possible to redirect participants’ behavior towards the proposed objectives by using reinforcement or punishment [24] (Skinner, 1988). The positive points could be used within the gamified intervention to modify the aesthetic of the avatar itself. However, such an avatar had no special abilities, but simply served to identify the participant. The avatars previously created by the teachers belonged only to the participant with whom the account was associated. The personalization of the avatar occurred randomly, allowing its modification by the students later spending their points. In ClassDojo the avatar initially appears as an egg. With the activation of the student account, the egg hatches showing the student’s avatar, which can be modified later. This gamified tool allows messages to be sent through the platform, either to inform students or legal guardians. Legal guardians may have an account associated with the student’s avatar in order to have greater control over the student’s behavior. Similarly, ClassDojo allows one to keep track of what happened in the sessions as a reminder. It also allows the grouping of avatars into teams, as it can receive positive or negative behavior points for all the team members for activities carried out as a team. To know the status of their points and how many they could spend, an updated score remained in view during the session, through an impression of points of each avatar, without a specific classification order, so they could not compare their progress according to the position in which they were placed. Students could check their score during recess, so at any moment the students knew their own and their team’s points. The gamified intervention was proposed using a PBL model (points-badges-leaderboards) with points that were distributed to avatars to be modified later or to score and acquire more points without showing any order among avatars; and an MDA model (mechanics-dynamics-aesthetics), through narrative about equipment, difficulty levels and perceived feelings, among others [12,13].

### 2.5. Statistical Analyses

The statistical program SPSS 24.0 was used for the analysis. Descriptive statistics (mean and standard deviation) of the variables to be measured were first analyzed. A Shapiro-Wilk test was carried out to check the sample normality, and found a non-normal distribution. Then, a Mann-Whitney U test was used to observe the initial differences between groups. Likewise, a Wilcoxon test was performed with the aim of observing longitudinal changes in the groups. Following Dominguez-Lara [25], the effect size was analyzed using Microsoft Excel software comparing the two groups according to rbis scores, using the standardized statistic of the Wilcoxon T (Z) and the total sample size (N). So, the following equation “Z/square root (N)” was used. The following cut points were used to observe the ES results, categorized into small (0.1–0.3), medium (0.3–0.5) and large size effects (>0.5) [26]. To analyze whether the changes produced in the EG were significant compared to those produced in the CG, a mixed-design analysis of variance was applied with two factors (time × method) and the effect size of the interaction calculated by eta squared. Furthermore, to check the reliability of the measuring instrument, Cronbach’s alpha analysis was performed detailed in the previous section on instruments (Section 2.3).

## 3. Results

### 3.1. Baseline Differences

The results from the Mann-Whitney U test (Table 2) did not show any statistical differences at baseline between EG and CG in any of the regulations measured, except for the variable introject in the total group. These results remain constant when the sample was segmented by sex.

### 3.2. Longitudinal Testing

The results from the Wilcoxon test (Table 3) present the changes within the same group after the didactic unit. On the one hand, the EG obtained a significant increase in intrinsic motivation (Z = −5.901; *p* < 001; ES = 0.373), identified regulation (Z = −5.901; *p* < 001; ES = 0.373), introjected regulation (Z = −5.901; *p* < 001; ES = 0.373) and external regulation (Z = −5.901; *p* < 001; ES = 0.373), however, amotivation did not show significant changes. On the other hand, there were no significant changes in the CG. The same results were observed when the sample was segmented by sex, regardless of the sex analyzed.

### 3.3. Hypothesis Testing

An interaction effect (Time × Method) was only observed for intrinsic motivation (F(1) = 5.263; *p* = 0.025; η^2^ = 0.070). This means that gamification in PE only improved intrinsic motivation significantly compared to the control group.

## 4. Discussion

The aim of the present work was to analyze the impact of a gamified intervention in elementary school PE classes compared to a non-gamified intervention on students’ motivation variables. The results showed increases in intrinsic motivation for EG compared to the CG, so H1 is accepted. Only one study found intrinsic motivation improvements when implementing a gamified intervention in elementary school students [19]. In addition, Quintas et al. [20] found improvements in BPNs, which are one of the causes of the emergence of intrinsic motivation [1]. In secondary education, other authors have also observed increases in intrinsic motivation in students when implementing various gamified didactics during sessions [6]. Similarly, Soriano-Pascual et al. [27] found improvements in BPNs, except for competence, relating to a better task climate and a decrease in disruptive behavior. These results may be due to the use of intrinsic rewards in the game and the promotion of BPNs through the game’s elements. However, other articles that investigated the effect of gamified learning in PE did not identify whether their motivational improvement achieved by gamification was intrinsic or not.

In parallel, increases in all the motivational regulations values that would indicate that extrinsic motivation did not increase compared to the CG was not observed, therefore, H2 is accepted. This agrees with Fernández-Río et al. [19] and Sotos-Martínez et al. [6], who found no change in extrinsic regulations. Nevertheless, in higher education there is research showing that a gamified learning program can achieve extrinsic increases [17,28]. This discrepancy in the results may be due to the nature of the rewards given in gamification as it appears that if the reward is intrinsic to the game it has a greater impact on intrinsic motivation, while regardless of whether it is more tangible or external, it affects extrinsic motivation [3,29]. In this way, the student could see that the rewards and punishments provided did not affect the educational process, only the development of the game raised through ClassDojo. Therefore, rewards and punishments did not provide extra help to the students in the educational process. On account of this, it is possible that students see rewards as useful tools for game development and relate them to the game itself, achieving an increase in intrinsic, but no change in extrinsic motivation. Although the age of students could be a significant relevant variable in this scenario, when students volunteer to participate in a gamified environment, one aspect repeated in many studies, with samples from primary to college students, is the student’s engagement with challenges [15,19,21,28,30,31,32,33,34,35,36].

Finally, the results showed no changes in the variable amotivation, hence H3 is rejected. This is contrary to what has been proposed by various authors, finding reductions in amotivation after applying a gamified proposal, both in elementary and secondary education [6,21]. Similarly, another research [18] did not find any change in amotivation after implanting a gamified intervention, however, it was observed that the gamified group tended towards a decrease in amotivation, while the CG showed increases in amotivation. Therefore, this lack of change in the variable amotivation is difficult to explain since, according to the SDT, an increase of intrinsic motivation should produce a decrease of amotivation [4]. Nevertheless, in the present study no change was observed. This lack of change could be due to several reasons. First, initial amotivation values (pre-test) were already very low in both treatment groups, so an expected change in this variable could be more difficult. Secondly, most studies [3,37,38] argue that an improvement in BPNs (autonomy, competence and relationship) leads to an improvement in intrinsic motivation. However, the design of the present intervention could have directly addressed the variables of autonomy and relationship (e.g., cooperative learning by grouping in teams or the possible perception of autonomy provided by immersion in the narrative) but less the competence of the students (e.g., use of scores, points they could use to compare their performance or avatars that were more modified), when a decrease in competence is directly related to an increase in amotivation [39,40,41]. An example of this would be the distribution of prizes or points, which were only awarded to students who performed the activity well, relating the development of their avatar in the game to this recognition and, thus, reducing the motivation of students to participate who did not acquire such positive recognition [38,42]. However, in this study, as indicated in the limitations, NPBs have not been evaluated, so more studies are needed to investigate this fact.

### Limitations and Strengths

The appearance of a greater novelty by participation in a new teaching method may have been conditioned on the increase in student motivation [43]. Similarly, the lack of measurement of NPBs is a factor that could have further clarified the results obtained. Another limitation is the lack of qualitative analysis that could augment the results found. The last limitation would be the sample size, which should be increased in future investigations. Finally, there is a shortage of studies covering this research topic in the educational stage of primary education making the present research important and relevant.

## 5. Conclusions

In the present research, a gamification with digital tools (ClassDojo^®^) based on 10 lessons produced an increase in intrinsic motivation in the experimental group when compared to the control group. However, there were no changes in extrinsic motivation and amotivation in the experimental group, as was expected from previous studies. Therefore, these results indicate that a gamified proposal in elementary school students of PE may improve the motivation of students, especially intrinsic regulation.

## Figures and Tables

**Table 1:** Description of items in the motivation questionnaire in physical education (CMEF-EP) [23].

ITEMS
Because PE is fun
2. Because I can learn skills that I could use in other areas of my life
3. Because I feel bad if I don’t participate in the activities
4. Because it is approved by the teacher and peers
5. But I don’t understand why we should have PE
6. Because I find this subject pleasant and interesting
7. Because I value the benefits that this subject has to develop as a person
8. Because I feel bad about myself if I miss class
9. Because I want the teacher to think that I am a good student
10. But I really feel like I’m wasting my time with this subject
11. Because I have fun doing activities
12. Because, for me, it’s one of the best ways to get useful skills for my future
13. Because I want my peers/sisters to value what I do
14. I do not know; my impression is that it is useless to continue attending class
15. For the satisfaction I feel when practicing
16. Because this subject gives me knowledge and skills that I consider important
17. To demonstrate to the teacher/colleague/s my interest in the subject
18. I don’t know clearly; because I don’t like anything

Intrinsic motivation = 1, 6, 11, 15; identified regulation = 2, 7, 12, 16; introjected regulation = 3, 8; external regulation = 4, 9, 13, 17; amotivation = 5, 10, 14, 18.

**Table 2:** Pre-test differences in different regulation variables between the CG and the EG (Av ± SD) in the entire sample and segmented by sex.

Sex	Variables	CG	EG	Sig.
Boys n_EG_ = 17 n_CG_ = 21	Intrinsic	4.25 ± 0.57	4.16 ± 0.55	0.592
	Identified	3.92 ± 0.76	3.85 ± 0.69	0.688
	Introjected	3.88 ± 1.13	3.38 ± 0.93	0.063
	External	3.52 ± 1.02	3.54 ± 0.95	0.733
	Amotivation	1.71 ± 0.76	1.65 ± 0.75	0.595
Girls n_EG_ = 18 n_CG_ = 16	Intrinsic	4.11 ± 0.71	4.40 ± 0.63	0.189
	Identified	4.08 ± 0.44	4.17 ± 0.69	0.639
	Introjected	3.41 ± 0.74	2.92 ± 1.26	0.225
	External	3.66 ± 0.59	3.10 ± 1.06	0.095
	Amotivation	1.64 ± 0.71	1.60 ± 0.58	1.000
Total n_EG_ = 35 n_CG_ = 37	Intrinsic	4.19 ± 0.63	4.29 ± 0.60	0.566
	Identified	3.99 ± 0.64	4.01 ± 0.70	0.973
	Introjected	3.68 ± 0.99	3.14 ± 1.12	0.023
	External	3.58 ± 0.85	3.31 ± 1.02	0.152
	Amotivation	1.68 ± 0.73	1.62 ± 0.65	0.702

CG = Control group; EG = Gamified group; Av = Average; SD = Standard Deviation; Sig = *p*-Value.

**Table 3:** Effects of the didactic unit for the CG and the EG in the different regulation motivational variables analyzed in the entire sample and segmented by sex (Av ± SD).

Sex	Variables	CG			EG			
		Pre Av ± SD	Post Av ± SD	Sig.	Pre Av ± SD	Post Av ± SD	Sig.	ES
Boys n_EG_ = 17 n_CG_ = 21	Intrinsic	4.25 ± 0.57	4.25 ± 0.61	0.952	4.16 ± 0.55	4.59 ± 0.34	0.005	0.476
	Identified	3.92 ± 0.76	3.90 ± 0.74	0.803	3.85 ± 0.69	4.22 ± 0.55	0.010	0.437
	Introjected	3.88 ± 1.13	3.81 ± 0.86	0.454	3.38 ± 0.93	3.76 ± 1.03	0.016	0.408
	External	3.52 ± 1.02	3.49 ± 0.99	0.470	3.54 ± 0.95	3.78 ± 0.97	0.027	0.374
	Amotivation	1.71 ± 0.76	1.67 ± 0.71	0.305	1.65 ± 0.75	1.54 ± 0.75	0.502	-
Girls n_EG_ = 18 n_CG_ = 16	Intrinsic	4.11 ± 0.71	4.17 ± 0.65	0.395	4.40 ± 0.63	4.78 ± 0.28	0.011	0.432
	Identified	4.08 ± 0.44	4.02 ± 0.51	0.157	4.17 ± 0.69	4.49 ± 0.41	0.019	0.397
	Introjected	3.41 ± 0.74	3.56 ± 0.44	0.129	2.92 ± 1.26	3.11 ± 1.27	0.035	0.357
	External	3.66 ± 0.59	3.66 ± 0.60	1.000	3.10 ± 1.06	3.38 ± 1.09	0.027	0.374
	Amotivation	1.64 ± 0.71	1.64 ± 0.71	0.705	1.60 ± 0.58	1.47 ± 0.50	0.122	-
Total n_EG_ = 35 n_CG_ = 37	Intrinsic	4.19 ± 0.63	4.22 ± 0.62	0.564	4.29 ± 0.60	4.69 ± 0.32	<0.001	0.633
	Identified	3.99 ± 0.64	3.95 ± 0.65	0.302	4.01 ± 0.70	4.36 ± 0.49	<0.001	0.589
	Introjected	3.68 ± 0.99	3.70 ± 0.71	0.646	3.14 ± 1.12	3.43 ± 1.19	0.001	0.544
	External	3.58 ± 0.85	3.56 ± 0.84	0.581	3.31 ± 1.02	3.57 ± 1.04	0.002	0.520
	Amotivation	1.68 ± 0.73	1.66 ± 0.70	0.343	1.62 ± 0.65	1.51 ± 0.62	0.120	-

CG = Control group; EG = Gamified group; Av = Average; SD = Standard Deviation; Sig = *p*-Value; ES = Effect Size.

## Data Availability

Not applicable.
